# Feasibility study of fluorescent lamp waste recycling by thermal desorption

**DOI:** 10.1007/s11356-021-16800-3

**Published:** 2021-10-05

**Authors:** José María Esbrí, Sofía Rivera, José Tejero, Pablo León Higueras

**Affiliations:** 1 Instituto de Geología Aplicada, Escuela de Ingeniería Minera e Industrial de Almadén, Universidad de Castilla-La Mancha, Plaza M. Meca 1, 13400 Almadén, Ciudad Real Spain; 2 Departamento de Mecánica Aplicada e Ingeniería de Proyectos, Universidad de Castilla-La Mancha, E.I.M.I. Almadén, 13400 Almadén, Ciudad Real Spain

**Keywords:** Hg, Thermal desorption, Fluorescent lamp waste, The Minamata Convention, Recycling, Rare earth elements

## Abstract

The proposed Minamata Convention ban on the use of fluorescent lamps at the end of 2020, with a consequent reduction in mercury (Hg) light products, is expected to produce large amounts of discarded fluorescent bulbs. In this context, the most effective recycling options are a thermal mercury recovery system and/or aqueous solution leaching (lixiviation) to recover rare earth elements (REEs). Due to the heterogeneous nature of these wastes, a complete characterization of Hg compounds in addition to a determination of their desorption temperatures is required for their recycling. The objective of this study is to assess the feasibility of a fast cost-effective thermal characterization to ameliorate recycling treatments. A pyrolysis heating system with a heat ramping capability combined with atomic absorption spectrometry makes it possible to obtain residue data with regard to the temperature ranges needed to achieve total Hg desorption. The major drawback of these heat treatments has been the amount of Hg absorbed from the residue by the glass matrices, ranging from 23.4 to 39.1% in the samples studied. Meanwhile, it has been estimated that 70% of Hg is recovered at a temperature of 437 °C.

## Introduction and background

The development of fluorescent lamps began in 1901 when Peter Cooper Hewitt invented the first mercury vapour lamp which emitted blue-green light. Nevertheless, the higher energy efficiency of these lamps led to further advances, culminating in the development by Germer, Meyer and Spanner in 1926 of tubes coated internally with fluorescent powder, which emitted white light. In 1980, the exterior design was modified to produce compact fluorescent lamps (CFLs).

A CFL consists of a copper (Cu) electrode, an aluminium (Al) cap, a tungsten (W) sub-electrode sealed in lead glass, a glass tube, fluorescent coating material, Hg, small amounts of argon (Ar) or krypton (Kr) and conductive Cu material (Lee et al. 2015). The fluorescent powder is basically composed of phosphorous compounds, most commonly derived from the following phosphates: Ca_5_(PO_4_)_3_(F_0.9_Cl_0.1_), Sr_5_(PO_4_)_3_(F_0.9_Cl_0.1_), Ca_5_(PO_4_)_3_(F, Cl): Sb^3+^, Mn^2+^ and (Ca, Sr)_10_(PO_4_)_6_(F, Cl)_2_: Sb^3+^, Mn^2+^ (Anand and Singh 2021). A wide variety of phosphorous compounds are used depending on the colour of light required. For example, the patented fluorescent lamp CN101307231A contains BaSiO_5_Pb (10–15%), 3Ca_3_(PO_4_)_2_Ca(F.Cl)_2_Sb (55–65%), MgO.MgF_2_CeO_28_Mn (5–10%) and (Zn.Sr)_3_(PO_4_)2Sn (20–25%) (Guosong 2008). In addition, rare earth elements (REEs), such as yttrium (Y), cerium (Ce), lanthanum (La), terbium (Tb) and europium (Eu), are commonly used (Anand and Singh 2021). Therefore, the recycling of this complex fluorescent light waste, containing large amounts of potentially toxic elements (PTEs) and REEs, can be economically viable but difficult to carry out.

The anthropogenic use of Hg worldwide, especially in its organic form, is highly toxic. Mercury, which has the capacity to travel long distances, is a global concern. In this context, the Minamata Convention (UNEP 2019) is aimed at protecting both the environment and human health against the adverse effects of mercury. Thus, technologies, such as fluorescent vapour lighting which uses and releases large quantities of Hg into the environment, were set to be banned by the end of 2020. These products, whose manufacture, export and import are prohibited, mostly concern the following: (i) general purpose CFLs (≤ 30 W) containing up to 5 mg Hg per lamp unit; (ii) linear fluorescent lamps (LFLs); (iii) high-pressure mercury vapour lamps (HPMVLs); and (iv) cold cathode fluorescent lamps (CCFLs) and external electrode fluorescent lamps (EEFLs).

The consequent reduction in the use of mercury in fluorescent lighting is expected to generate large amounts of discarded products. Most of this waste will be deposited in landfills, along with other household waste, with only a small proportion being recycled: around 4–7% in Canada, 23% in the USA, 6% in Brazil, 80% in Austria and 95% in Switzerland (de Farias et al. 2020; Silveira and Chang 2011). In Europe, waste electrical and electronic equipment (WEEE) directive 202/96/CE recommends a minimum recovery rate of 70%, as well as a reuse and recycling rate of over 50%, which will be technologically challenging to achieve.

CFLs can be recycled through the use of either thermal or chemical processes involving aqueous lixiviation (Durao et al. 2008). Generally, thermal desorption systems expose waste to high temperatures up to boiling point to recover mercury which is then converted into condensed vapour. Some systems also include a purification stage involving distillation or nitric acid bubbling processes. Several thermal desorption methods have been developed: Sweden’s mercury recovery technology (MRT) system; end-of-life fluorescent lamp recycling technology developed by Nomura Kohsan in Japan; and the AERC and SepaDyne mercury recycling facilities in the USA (Rodríguez et al. 2012; Lee et al. 2015; Morris et al. 2002; TRU and Mixed Waste Focus Area 2002; Weyand et al. 1995). The most common recycling method used in Spain is based on the Swedish MRT system (Chang et al. 2007). This method includes an initial metal cap separation process, followed by the collection of fluorescent powder from the tubes, a glass crushing stage and Hg recovery involving distillation at temperatures of over 375 °C (Lee et al. 2015). These operational temperatures could be underestimates, as this type of waste normally requires temperatures of between 600 and 800 °C to ensure complete Hg removal due to the presence of Hg oxides when desorption temperatures exceed 375 °C (Raposo et al. 2003). The whole process is performed under negative pressure conditions to prevent the release of Hg emissions into the atmosphere.

As the principal drawback of thermal treatments has always been their high cost, an appropriate balance between efficiency, temperature and treatment period needs to be found in order to make the process more cost-effective. The determination of minimum desorption temperatures depends on the heterogeneous nature of CFL residues. This study aims to find a cost-effective method to evaluate the optimum minimum temperature range for each CFL waste product in order to reduce total mercury content to below the legal limits laid down by the European Union directives.

## Experimental section

Sampling was carried out over 5 consecutive days in May 2017. Each composite sample, weighing a total of 3 kg and containing CFL residues, was crushed in the recycling facility and then homogenized and mixed, prior to the extraction of an aliquot of 100 g, and finally ground in an agate mortar for 5 min to ensure appropriate grain size.

A multielemental characterization was carried out with the aid of an Epsilon1 energy-dispersive X-ray fluorescence analyzer and the PanAlytical software. This enabled us to carry out four consecutive energy determinations of groups of elements from sodium (Na) to americium (Am) with similar matrix effects. Major and trace elements were quantified after editing the fluorescence spectra which were then quantified using the appropriate method, with the number of fluorescence signals detected for each element being changed accordingly. Sample analysis was carried out in duplicate to ensure representative results.

Total Hg and Hg compounds in the CFL residues were analysed using the Zeeman atomic absorption spectrometer (ZAAS) Lumex RA-915 M attached to a PYRO-915 + atomizer (Sholupov et al. 2004). Total Hg data were obtained by pyrolyzing each sample at 800 °C in order to atomize the Hg compounds. With its low detection limit (2 ng g^−1^), a high quantitative determination capacity of up to 2% and, due to the Zeeman effect, the negligible effect of matrices on the final results, this is the most effective technique for analysing these types of samples. Quality control was carried out by analysing certified reference material (NIST 2710a), with recovery rates of 98.5–107.2% being achieved.

Mercury speciation studies were carried out using a Lumex RA-915 M device coupled with a PYRO-915 + pyrolysis device, and specific temperature ramp software (Rumayor et al. 2016). This equipment enables different voltages, electrical resistances and time periods to be selected.

## Results and discussion

### Multielemental and Total Hg characterization

Multielement concentrations showed a predominance of silicon dioxide (SiO_2_) in CFL residues, accounting for an average of 32.77% of the total, while CaO accounted for 15.13% and Al_2_O_3_ and P_2_O_5_ 8.89% and 8.77%, respectively (Table 1). In addition, REE concentrations reached significant levels, with yttrium (Y) accounting for 6.77%, and cerium (Ce), europium (Eu), terbium (Tb) and lanthanum (La) representing only 0.57%, 0.43%, 0.38% and 0.34%, respectively, of the total. Trace elements included sulphur trioxide (SO_3_; 835 mg kg^−1^), titanium (Ti; 704 mg kg^−1^), zinc (Zn; 580 mg kg^−1^) and tungsten (W; 541 mg kg^−1^), as well as small amounts of copper (Cu), tin (Sn) and zirconium (Zr). The fluorescent tube glass in these residues, which has not been separated from the phosphorous powder, is thus a raw sample of the crushed tube. Cu concentrations are sufficiently low to rule out the presence of electrical parts of the tube in the residues. While these results accord with other CFL wastes (de Farias et al. 2020; Raposo et al. 2003), some residues contain phosphor powder while others contain glass bulbs. Some studies have identified the main elements, Ca, P, Sr, Na, Ba, Mg, Sb, K, Mn, Fe and Hg, in phosphor powder, with varying amounts of REEs such as Y, Eu, Ce, Gd and La (Pavón et al. 2021); thus, tubes and other lamp parts contained the elements Al_2_O_3_, SiO_2_ and Pb, as well as most trace elements. In addition, while REEs such as Tb were present in the waste studied, Gd was not found.

**Table 1:** Statistical summary of multielement concentrations of CFL waste samples, expressed as a % in the left column and in mg kg^−1^ in the right column. Hg concentrations were analysed using ZAAS

Element	Mass (%)	SD	Element	Mass (mg kg^−1^)	SD
Al_2_O_3_	8.89	1.09	SO_3_	835.58	109.99
SiO_2_	32.27	1.39	Ti	704.10	125.43
P_2_O_5_	8.77	0.41	Co	43.72	61.45
Cl	0.36	0.01	Ni	59.90	47.32
K_2_O	1.19	0.19	Cu	312.90	41.01
CaO	15.13	0.97	Zn	580.00	87.83
Mn	0.13	0.01	Ge	108.48	17.01
Fe_2_O_3_	1.25	0.06	Te	113.72	14.43
Sb	0.14	0.01	W	541.06	90.85
Ba	1.28	0.18	Hg	139.72	9.74
La	0.34	0.02	Th	4.56	5.06
Ce	0.57	0.14	As	126.10	60.88
Pb	0.42	0.07	Br	174.24	61.97
Eu	0.43	0.14	Rb	40.06	5.51
Tb	0.38	0.10	Zr	243.24	21.14
Sr	0.39	0.02	Sn	344.88	57.30
Y	6.77	1.99			

Total Hg (THg) in CFL samples ranged from 125.4 to 151.3 mg kg^−1^, with a very low variation coefficient of 6.9% observed over the 5 sampling days. These figures are much lower than those for spent CFL bulbs in other studies: 1430–13,300 mg kg^−1^ (Raposo et al. 2003) and 3418.34 ± 269.26 mg kg^−1^ (Choi and Rhee 2017). Considerable Hg concentrations can be found in fluorescent glass bulbs, ranging greatly around the world from 8.27% (Jang et al. 2005), 13.66% (Rey-Raap and Gallardo 2013) and 16.27% (dos Santos et al. 2010) to 21.6% (Taghipour et al. 2014) and 50% (Hobohm et al. 2017). Thus, the CFL residue used in this study can be regarded as highly homogeneous, while the Hg content of different residues worldwide varies greatly, with, in addition, major differences in matrix/fractionation effects. This heterogeneity on a global scale is an enormous obstacle to implementing generic recycling techniques for this type of waste. Thus, in order to adapt these techniques to the multielement contents of each CFL residue, it is essential to characterize various aspects of the waste, including total Hg and its matrix/fractionation effects. The use of thermal desorption is particularly recommended for rapid low-cost evaluation of these two factors. This study evaluates the feasibility of thermal speciation of mercury with the aid of a Lumex RA-915 M analyzer.

### Thermal speciation of Hg

**Fig. 1:**
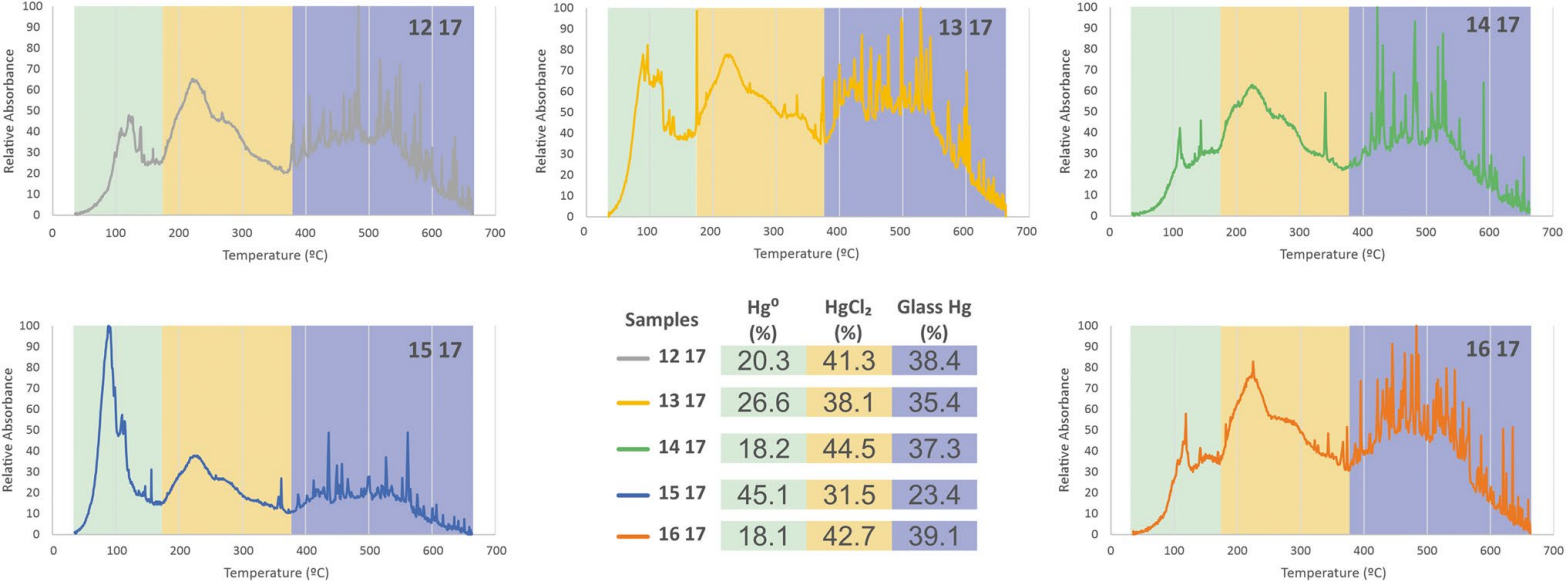
Thermal speciation Hg profiles of CFL samples, showing desorption peaks of Hg0 (in green), Hg chlorides (in orange) and Hg adsorption by glass tubes (in blue). Relative proportions of these Hg compounds are shown in percentages

The thermal desorption profiles of five samples in Fig. 1 show three clearly delimited Hg species: elemental Hg (Hg^0^), released at 35–174 °C; HgCl_2_/Hg_2_Cl_2_, released at 174–367 °C; and glass Hg, released at 367–662 °C. In fluorescent tubes, Hg is usually found in its elemental Hg^0^ form, which is ionized each time the light is switched on. The Hg compounds formed are therefore considered simple combinations of Hg^0^, Hg^+1^ and/or Hg^+2^, with the most accessible compounds, in this case mainly Cl, having more affinity to Hg. A similar analysis of phosphor powder from spent fluorescent tubes in Brazil has demonstrated the presence of four main species: Hg^0^, HgCl_2_, Hg_2_Cl_2_ and HgO (Raposo et al. 2003). However, this identification of Hg compounds cannot be regarded as fool proof. In some ways, the thermal desorption method for identifying Hg species is similar to the X-ray diffraction method for the identification of mineral phases, which also produces analytical results that need to be interpreted by an experienced researcher who is familiar with the samples. Mercury compounds are desorbed at different temperatures, some of whose desorption ranges can overlap considerably. This is the case for many compounds, such as HgBr, HgI, HgCl_2_ and Hg_2_Cl_2_, as well as humic acid-bound and oxyhydroxide-bound Hg fractions, which desorb at around 200–225 °C. No humic acid-bound Hg has been found in discarded fluorescent bulbs despite the considerable information available on chemical elements (Table 1). For example, although there is sufficient S for metacinnabar formation, X-ray fluorescence (XRF) analysis does not provide sufficiently clear data on fluorescent light elements capable of forming compounds with the Hg species Hg^0^, Hg^+1^ and Hg^2+^ present in discarded fluorescent tubes. Although the identification of the majority of species identified was based on data provided by the same technique (Raposo et al. 2003), our study sees the frequent peaks of the Hg species Hg^2+^ in desorption profiles as caused by the crushing of material containing Hg, which is a typical cinnabar desorption profile from a geological sample. In addition, HgO is regarded as incapable of producing these successive peaks, suggesting a continuous crushing of structured material containing Hg.

**Fig. 2:**
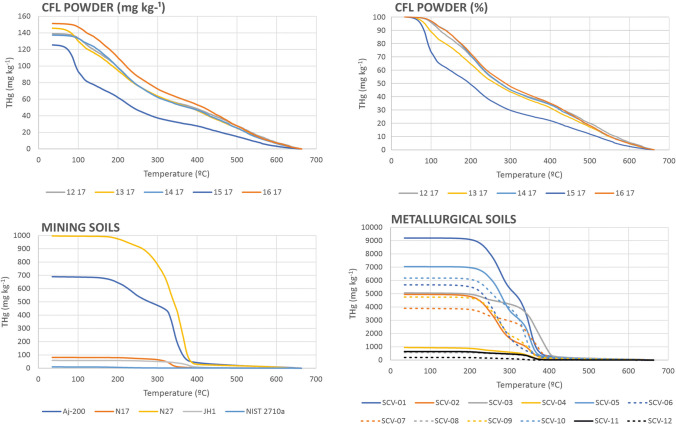
Comparison of thermal desorption Hg profiles of some Hg waste products: CFL samples, as well as mining and metallurgical soils

Nevertheless, the usefulness of these desorption graphs (Fig. 1) lies in the delimited temperature ranges, within which each compound is desorbed, especially those desorbed at above 375 °C, at which all Hg is extracted from the sample using a traditional recycling system for this type of waste material. Also, species fractionation showed that the samples studies are highly homogeneous except for the sample 15 17, which with its higher Hg^0^ content does not maintain the proportion of species. It is important to note that 35–40% of total Hg in discarded fluorescent tubes is adsorbed to glass. When the desorption temperature for this Hg compound was 367 °C at the beginning of the process, the residues still contained 60 mg kg^−1^, or about 38% of THg at the initial stage of pyrolysis (Fig. 2). Like soils contaminated by mining and metallurgical activities, other residues desorb most of their Hg at 375 °C (Fig. 2). The samples come from contaminated mining soil in Almadenejos (Aj-200) and Almadén (N17 and N27) in Spain, the Czech Republic (JH1) and the USA (NIST 2710a); as well as metallurgical sites in Almadenejos (SCV samples). Speciation data for these samples show Hg compounds with desorption temperatures below the abovementioned limit of 375 °C, which are similar to those for metacinnabar (280 °C), methyl Hg (290 °C), humic acid-bound Hg (220 °C) and cinnabar (305 °C) (Campos et al. 2018).

CFL recycling regulations outlined in directive 202/96/CE lay down a minimum recovery rate of 70%, as well as a reuse and recycling rate of over 50% for CFL components, material and substances. This means that a 70% minimum recovery rate is mandatory for Hg in CFL residues, which requires a temperature of 437 °C to be reached in CFL samples (Fig. 2).

### Optimization of thermal desorption technique

In order to determine the minimum working temperature in a recycling furnace to extract the highest possible amount of mercury from samples, an initial temperature ramp of 509 °C was selected. The efficiency of this heating ramp was verified using cinnabar, a naturally occurring mercuric sulphide (HgS) from the Almadén mine in Spain, milled to a grain size of less than 70 microns and diluted in quartz powder. Maximum temperatures corresponded to the typical desorption temperature of 305 °C for cinnabar and 400 °C for Hg oxides in phosphorus powder (Rumayor et al. 2016). During this process of optimization, several factors were found to affect the precise determination of mercury desorption temperatures: the grain size of the sample, which should not exceed 100 microns; electrical resistance/voltage changes, which should not lead to rapid heating of samples that would increase desorption; the position and quantity of the sample in the boat (sample holder), which should be systematically regulated; and air flow which was enhanced at higher rates (3 L min^−1^).

Most critical parameters were flow rate and homogenization of heat ramp. To optimize these parameters, some tests were carried out with different flow rates and different degrees of homogenization of the heating ramps.

**Fig. 3:**
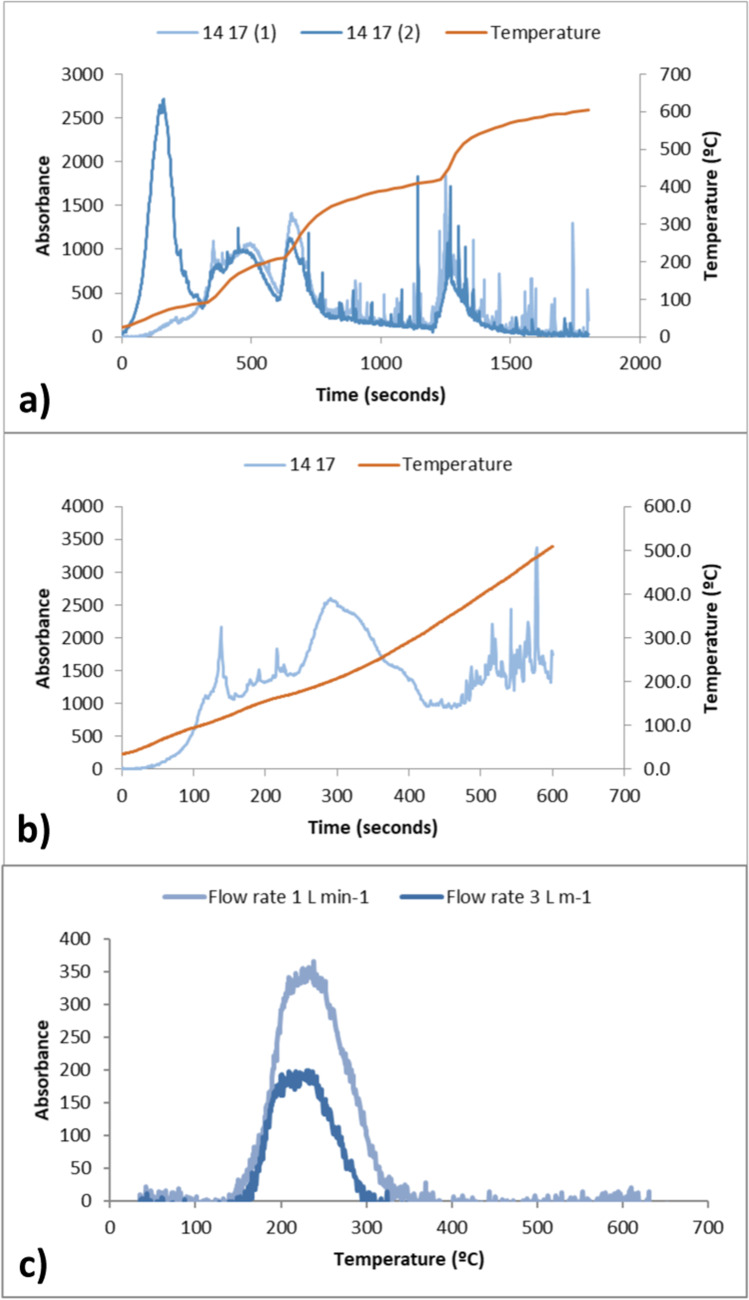
Desorption profiles of method optimization. **a** and **b** Optimization of heat ramp. **c** Optimization of flow rate

In the equipment used in this work, the heating ramp is designed by assigning increasing voltages to the different units of the pyrolyzer. The first heating ramps smoothed out long desorption times and had too abrupt voltage jumps. This produced an increase in desorbed Hg coinciding with the voltage jumps of the heating ramp. This effect can be perfectly observed in Fig. 3a, in which the onset of several desorption peaks coincides with the rapid increase in temperature. To soften this effect and design a homogeneous heating ramp in terms of temperature, two parameters were modified, the magnitude of the voltage increase steps and the total time, reducing both until achieving a linear ramp with no effect on the desorption peaks (Fig. 3b).

Figure 3c shows how the flow rate affects the recorded desorption temperature. An evident delay is observed between the sample that has been desorbed at a lower flow rate (1 L min^−1^) and the one that desorbed at 3 L min^−1^. This is most probably due to the length of the mercury path between the second unit of the pyrolyzer and the analytical cell, which in the design of this experiment is located in the high concentration cell, located outside the pyrolyzer, at the backside of the Lumex RA-915 M mercury analyzer. No improvement in peak definition has been observed, although it is an expected effect of high flow rates.

Finally, the configuration was optimized under the following conditions:

- Heating rate: 0.74 °C s^−1^
- Heating range: 35–660 °C
- Sample mass: 20 mg
- Pyrolyzer air flow: 3 L min^−1^
- Cooling time intervals between successive determinations: 40 min.

Using this configuration, incomplete desorption profiles were obtained, with over 20% of Hg still not released. The desorption profiles showed that the samples released mercury at the temperatures of Hg oxides, although another more refractory Hg compound remained unreleased. To resolve this problem, the temperature was ramped up to 660 °C.

### Discussion

Crushed fluorescent lamp waste, which is a mixture of phosphorous powder and crushed glass with very different levels of Hg content and other elements, including highly valuable REEs, can be considered a complex residue in terms of recycling capacity. The recycling strategy for these wastes currently combines a prior Hg thermal release stage and a subsequent leaching stage for usable elements, mainly REEs. This study, as well as others (Jang et al. 2005; Rey-Raap and Gallardo 2013; Taghipour et al. 2014; Hobohm et al. 2017), highlights the large amounts of Hg trapped in glass, which requires a high temperature of 437 °C to achieve a recovery rate of at least 70%. Some research describes the impact of thermal recovery treatment times on these waste products (Choi and Rhee 2017), with an optimal treatment time of 360 min at 400 °C for phosphorous powder using the reaction rate constant and activation energy. These promising results are dependent on the fractionation of Hg compounds in waste, especially those trapped in glass. It is important to note that CFL residues show large compositional differences between countries and between fluorescent lamp recycling companies (Binnemans et al. 2013), and that REEs are often recovered following the Hg recovery stage. Thus, a comprehensive and rapid characterization of mercury glass fractions is required in order to carry out thermal recovery treatments of such heterogeneous waste materials.

## Conclusions

The restrictions proposed by the Minamata Convention (UNEP 2019) will lead to large quantities of waste containing Hg and REEs. The 2030 Agenda for Sustainable Development (United Nations 2015) calls on the world to “upgrade infrastructure and retrofit industries to make them sustainable, with increased resource-use efficiency and greater adoption of clean and environmentally technologies” by 2030 (Goal 9). This means that the problem of heterogeneous CFL waste could be resolved in a sustainable manner by combining two recycling methods in order to eliminate mercury and to recover REEs.

This study describes a fast, low-cost thermal mercury speciation system to accurately determine optimal desorption temperatures for CFL residues using atomic absorption spectrometry.

Although the study describes the combined use of a Lumex RA-915 device, a pyrolysis device and an atomic absorption spectrometer determine the desorption temperatures on an industrial scale.

We recommend the following conditions:

- Continuous heating from 35 to 660 °C, while avoiding accelerations that could cause early Hg desorption.
- A high flow rate of 3 L min^−1^ to ensure more accurate determination of desorption temperatures.
- A heat rate of 0.74 °C s^−1^.
- Cooling time of 40 min to ensure an appropriate starting temperature.

## Data Availability

José María Esbrí, researcher, PhD, as responsible of the manuscript entitled “Feasibility study of fluorescent lamp waste recycling by thermal desorption”, authored by myself and Sofía Rivera-Jurado, MsC, José Tejero, PhD and Prof. Pablo Higueras, PhD. On behalf of the rest of the coauthors, with this document I warrantee and sign that the datasets generated and used during the current study are available from the corresponding author on reasonable request.

## References

[CR1] Anand A, Singh R (2021). Synthesis of rare earth compounds from phosphor coating of spent fluorescent lamps. Sep Purif Rev.

[CR2] Binnemans K, Jones PT, Blanpain B, Van Gerven T, Yang Y, Walton A, Buchert M (2013). Recycling of rare earths: separation & purification reviews 15 A Critical Review. J Clean Prod.

[CR3] Campos JA, Esbrí JM, Madrid MM, Naharro R, Peco J, García-Noguero EM, Amorós JA, Higueras P (2018). Does mercury presence in soils promote their microbial activity? The Almadenejos case (Almadén mercury mining district, Spain). Chemosphere.

[CR4] Chang TC, You SJ, Yu BS, Kong HW (2007). The fate and management of high mercury-containing lamps from high technology industry. J Hazard Mater.

[CR5] Choi Y, Rhee S (2017). Evaluation of energy consumption in the mercury treatment of phosphor powder from spent fluorescent lamps using a thermal process. Sustainability (Switzerland).

[CR6] de Farias CV, Paulino JF, Barcelos DA, Rodrigues APDC, Pontes FVM (2020) Is mercury in fluorescent lamps the only risk to human health? A study of environmental mobility of toxic metals and human health risk assessment. Chemosphere 261.10.1016/j.chemosphere.2020.128107 10.1016/j.chemosphere.2020.128107 33113668

[CR7] dos Santos EJ, Herrmann AB, Vieira F, Sato CS, Correa QB, Maranhao TA, Tormen L, Curtius AJ (2010). Determination of Hg and Pb in compact fluorescent lamp by slurry sampling inductively coupled plasma optical emission spectrometry. Microchem J.

[CR8] Durao WA, de Castro CA, Windmoller CC (2008). Mercury reduction studies to facilitate the thermal decontamination of phosphor powder residues from spent fluorescent lamps. Waste Manag.

[CR9] Guosong Z Patent CN101307231A (2008) Fluorescent powder composition. Available online: https://patents.google.com/patent/CN101307231A/en. Accessed on 26 Jan 2021

[CR10] Hobohm J, Krüger O, Basu S, Kuchta K, van Wasen S, Adam C (2017). Recycling oriented comparison of mercury distribution in new and spent fluorescent lamps and their potential risk. Chemosphere.

[CR11] Jang M, Hong SM, Park JK (2005). Characterization and recovery of mercury from spent fluorescent lamps. Waste Manag.

[CR12] Lee C, Popuri SR, Peng Y, Fang S, Lin K, Fan K, Chang T (2015). Overview on industrial recycling technologies and management strategies of end-of-life fluorescent lamps in Taiwan and other developed countries. J Mater Cycles Waste Manag.

[CR13] Morris MI, Osborne-Lee IW, Hulet GA (2002) Demonstration of new technologies required for the treatment of mixed waste contaminated with ≥260 ppm mercury, U.S. Department of Energy Office of Science and Technology, 2002

[CR14] Pavón S, Lorenz T, Fortuny A, Sastre AM, Bertau M (2021). Rare earth elements recovery from secondary wastes by solid-state chlorination and selective organic leaching. Waste Manag.

[CR15] Raposo C, CarvalhinhoWindmöller C, DurãoJúnior WA (2003). Mercury speciation in fluorescent lamps by thermal release analysis. Waste Manag.

[CR16] Rey-Raap N, Gallardo A (2013). Removal of mercury bonded in residual glass from spent fluorescent lamps. J Environ Manag.

[CR17] Rodríguez O, Padilla I, Tayibi H, López-Delgado A (2012). Concerns on liquid mercury and mercury-containing wastes: a review of the treatment technologies for the safe storage. J Environ Manag.

[CR18] Rumayor M, Lopez-Anton MA, Díaz-Somoano M, Maroto-Valer MM, Richard J, Biester H, Martínez-Tarazona MR (2016). A comparison of devices using thermal desorption for mercury speciation in solids. Talanta.

[CR19] Sholupov S, Pogarev S, Ryzhov V, Mashyanov N, Stroganov A (2004). Zeeman atomic absorption spectrometer RA-915+ for direct determination of mercury in air and complex matrix samples. Fuel Process Technol.

[CR20] Silveira GTR, Chang SY (2011). Fluorescent lamp recycling initiatives in the United States and a recycling proposal based on extended producer responsibility and product stewardship concepts. Waste Manag Res.

[CR21] Taghipour H, Amjad Z, Jafarabadi MA, Gholampour A, Nowrouz P (2014). Determining heavy metals in spent compact fluorescent lamps (CFLs) and their waste management challenges: some strategies for improving current conditions. Waste Manag..

[CR22] TRU and Mixed Waste Focus Area (2002) The SepraDyneTM-raduce system for recovery of mercury from mixed waste, New York: Innovative Technology Summary Report, September, 2002

[CR23] United Nations (2015) Transforming our world: the 2030 Agenda for Sustainable Development. Main Committee (A/70/L.1). Available at https://www.un.org/ga/search/view_doc.asp?symbol=A/RES/70/1&Lang=E. Accessed 14/05/2021

[CR24] UNEP (2019) Minamata Convention on Mercury. Text and annexes. Available online: https://www.mercuryconvention.org/Portals/11/documents/Booklets/COP3-version/Minamata-Convention-booklet-Sep2019-EN.pdf. Accessed on 26 Jan 2021

[CR25] Weyand TE, Zugates TP, Rose MV (1995) Performance of MRS’s commercial medium-temperature mercury desorption process. In: International Incineration Conference, Bellevue, WA, U.S.A., May, 1995

